# Evaluation of SNP calling using single and multiple-sample calling algorithms by validation against array base genotyping and Mendelian inheritance

**DOI:** 10.1186/1756-0500-7-747

**Published:** 2014-10-22

**Authors:** Pankaj Kumar, Mashael Al-Shafai, Wadha Ahmed Al Muftah, Nader Chalhoub, Mahmoud F Elsaid, Alice Abdel Aleem, Karsten Suhre

**Affiliations:** Weill Cornell Medical College in Qatar, Education City, Doha, Qatar; Neuropediatrics Department, Hamad Medical Corporation, Doha, Qatar; Institute of Bioinformatics and System Biology, Helmholtz Zentrum Munchen, German Research Center of Environmental Health, Nuherberg, Germany

**Keywords:** NGS, GATK, CASAVA, WGS pipeline, Mendelian inheritance, Qatari population, Multi-sample calling, Genotype calling, Variant, Trios, Illumina

## Abstract

**Background:**

With diminishing costs of next generation sequencing (NGS), whole genome analysis becomes a standard tool for identifying genetic causes of inherited diseases. Commercial NGS service providers in general not only provide raw genomic reads, but further deliver SNP calls to their clients. However, the question for the user arises whether to use the SNP data as is, or process the raw sequencing data further through more sophisticated SNP calling pipelines with more advanced algorithms.

**Results:**

Here we report a detailed comparison of SNPs called using the popular GATK multiple-sample calling protocol to SNPs delivered as part of a 40x whole genome sequencing project by Illumina Inc of 171 human genomes of Arab descent (108 unrelated Qatari genomes, 19 trios, and 2 families with rare diseases) and compare them to variants provided by the Illumina CASAVA pipeline. GATK multi-sample calling identifies more variants than the CASAVA pipeline. The additional variants from GATK are robust for Mendelian consistencies but weak in terms of statistical parameters such as TsTv ratio. However, these additional variants do not make a difference in detecting the causative variants in the studied phenotype.

**Conclusion:**

Both pipelines, GATK multi-sample calling and Illumina CASAVA single sample calling, have highly similar performance in SNP calling at the level of putatively causative variants.

**Electronic supplementary material:**

The online version of this article (doi:10.1186/1756-0500-7-747) contains supplementary material, which is available to authorized users.

## Background

Numerous NGS pipelines and tools have been developed in recent years that are valuable to users in the field, but also create confusion in selecting the desired tool. Some of the commercial NGS pipelines are CLC genomics workbench, DNASTAR, CASAVA, Genious, Genomatix Solutions, GenoMiner, Partek Genomics Suite and so on. Most of the commercial NGS pipeline tools are targeted to biologists as end-users highlighting easy and user friendly interface. Often, these commercial tools become difficult to customize for speed when processing large number of samples. Alternatively, commercial vendors provide the facility to process and ship the complete variants sets along with the sequencing of samples. Non-commercial open source NGS pipelines such as GATK [1, 2], SAMtools [3], SOAP [4, 5], SNPAAMapper [6], WEP [7], Atlas2 [8] are also being used extensively in academia and many organizations. These open source NGS pipelines are highly customizable but require expertise to set up optimally. Many studies have been done to evaluate NGS data analysis pipelines and tools. Bao S. et al. [9] have evaluated various mapping and assembly software. Pabinger et al. [10] have surveyed around 205 variants of NGS tools at different analytical steps like quality assessment, alignment, variant identification, variant annotation and visualization. Nielsen et al. [11] have evaluated various SNP and genotype calling algorithms. Although these studies have helped tremendously in determining which tools and pipelines to use, they do not answer the concrete question of whether to use data provided from a commercial vendor or to put in extra efforts to run additional well-known open source pipelines. Also, in situations where we fail to identify a causative variant in the data set provided by commercial vendors, we may doubt the pipeline’s ability to find the variants. Thus, it becomes important to compare the variant sets provided by commercial vendors with variants obtained through one of the well-reputed tools. Several studies have confirmed the GATK pipeline’s excellent performance in detecting variants. The GATK pipeline is being used in large projects, such as the 1000 Genomes Project and The Cancer Genome Atlas [1, 12]. However, smaller labs and institutes often rely fully on commercial vendors for complete sequencing and analysis services. Illumina Inc. is a leader in providing NGS services. Illumina uses the CASAVA and ISSAC pipelines for variant detection. Illumina has reported comparison among ISAAC, CASAVA and GATK pipelines; mostly for the speed of completing the pipeline [13]. However, an independent detailed comparison between the Illumina and GATK pipeline using multi-sample calling algorithm in larger cohorts is necessary. Here we compared variant sets supplied by the Illumina CASAVA pipeline and the well-known GATK pipelines in great detail on concrete study cases and discuss the differences from a user’s perspective. In general, genotype calling errors by the variant callers are associated with Mendelian violation when the caller is unaware of family structure [14]. In this study, both GATK and CASAVA are unaware of family pedigree and therefore Mendelian inheritance is examined in familial samples for the genotypes of discordant variants by the pipelines. As an additional independent quality control we use genotyping array data from the Illumina OMNI 2.5 array. We present an evaluation of the CASAVA and the GATK pipelines for three different data sets: 108 unrelated Qatari genomes, 19 trios from studies on obesity and diabetes, and 2 larger families with suspected rare genetic diseases.

## Methods

### CASSAVA SNP calling

Illumina SNP calls were based on the CASAVA -1.9.0a1_110909 pipeline. SNPs and the genotype from the CASAVA pipeline were called for each sample individually. We created a pass quality subset of these variants by keeping the variant for which Filter column in VCF file has value "PASS" and removing all other variants. Thus, the first set without any quality filter will be called CASAVA ALL and the quality filtered set will be called CASAVA PASS in this paper. In many cases, we have compared the pipelines for a group of samples. In these cases, we merged these SNPs from the CASAVA pipeline using vcftools [15]. Similarly, we created merged VCF for quality filtered (PASS quality) from the CASAVA pipeline by merging all the PASS quality SNPs based on quality column annotation (Genotype quality >20) in all single sample VCF files.

### GATK best practice pipeline

In our in-house pipeline, we used Bowtie2 [16] to align the sequencing reads against the human reference genome build 37. We also used other necessary tools like SAMtools [3], Novosort and Picard [17] to process and format alignment files before processing them with GATK. We implemented the best practices of GATK pipeline to call SNPs and Indels. We have used GATK 2.4 version and GATK-UnifiedGenotyper as SNP caller in this study. We have used multi-sample variant calling by GATK-UnifiedGenotyper. The reason of using multi-sample calling is to distinguish non-variant genotypes between homozygous reference genotype and missing genotype in cohort analysis. With single sample calling genotype called only for variants we can’t be sure if the non-variants have missing genotype or same as reference. Also, big projects like 1000 genomes have preferred multi-sample calling over single sample calling [18]. We used GATK-UnifiedGenotyper instead of GATK-HaplotypeCaller, a similar or better variant caller by GATK, in this study because of similar accuracy in calling SNPs and computational feasibility to run for large number of samples. For more than 100 samples, according to GATK website, GATK-UnifiedGenotyper is advised over GATK-HaplotypeCaller. The real advantage of Haplotypecaller over UnifiedGenotyper is in calling Indels but in this paper we are focusing on SNPs only. Next, similar to the CASAVA pipeline, we created two variant sets, GATK ALL (without any quality filter) and GATK PASS (by keeping the variant for which Filter column in VCF file has value "PASS" and removing all other variants) from our in-house GATK pipeline. The variants found by GATK pipeline were recalibrated using GATK walker VariantScoreRecalibrater. The input true sites in creating the model were SNPs from dbSNP Human build 132 [19], genotyping OMNI array calls of 1000 genomes project and Hapmap SNP calls for estimating the probability that SNPs are true genetic variants rather than a sequencing or data processing artifact. The call sets were partitioned into quality trenches and are shown in the plot below. We took the variants until we found 99% of known variants (truth sensitivity) in the GATK PASS variant set.

### Genotyping Omni array

Human genotyping array data is from Illumina HumanOmni2.5-8 platform. This array has about 2.37 million tag SNPs from 1000 genomes pilot project with MAF ≥2.5%. Illumina Inc. supplied genotypes for all the samples from HumanOmni2.5-8 by performing Illumina Infinium LCG assay and thereupon calling the genotypes using their propriety software called GenomeStudio. They provide genotype for each of these probes with GenCall scores. Illumina recommends a GenCall score cut-off of 0.15 for their infinium assay based products [20]. This recommended GenCall score cut-off of 0.15 was used to test the concordance with the GATK and CASAVA pipelines.

### Evaluation data sets

**Figure 1:**
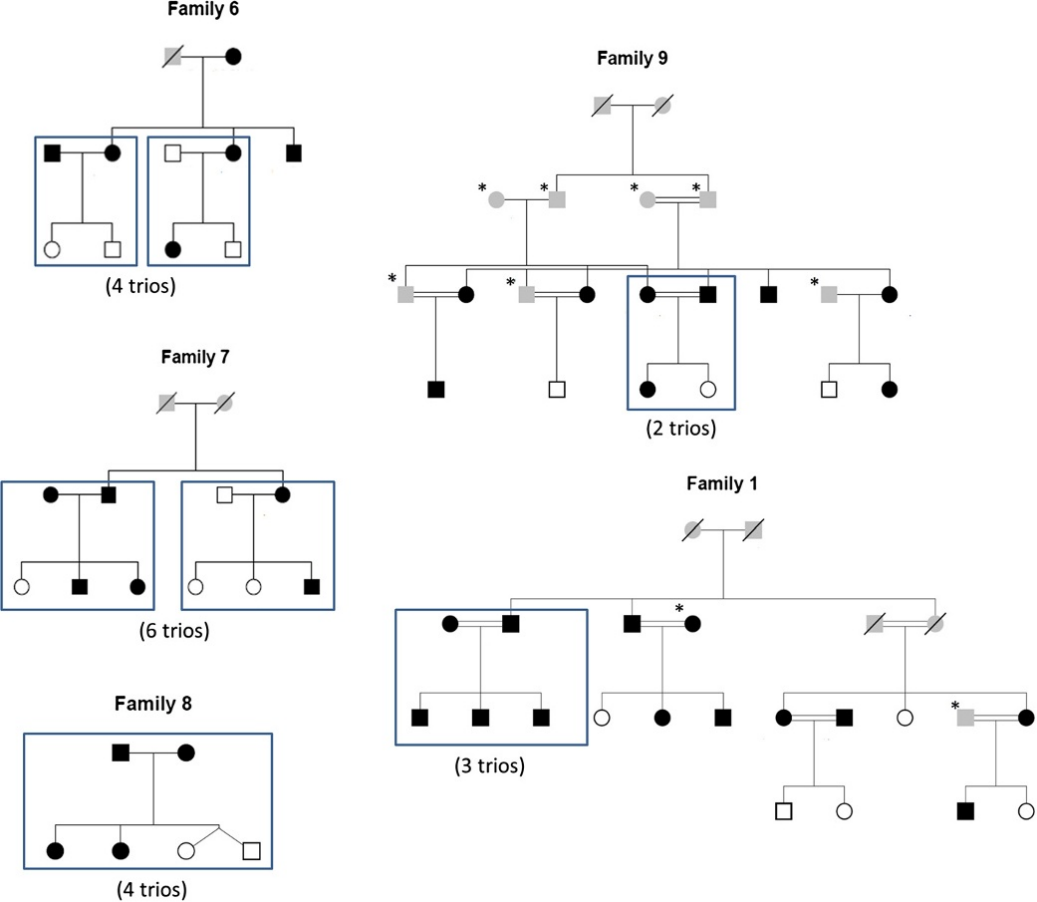
**19 trios in family pedigree.** Rectangular boxes drawn in the family pedigree indicate the trios taken for pipelines comparison analysis. Individual in black are obese. Star marked individuals were not sequenced. Individuals greyed in the pedigrees had unknown phenotype. Individual with no color are non-obese individuals.

**Figure 2:**
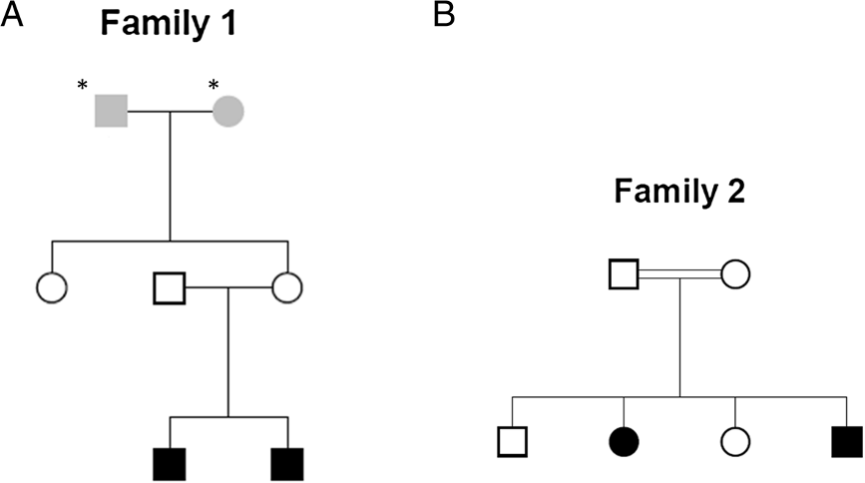
**Pedigrees of the families with children affected with monogenic homozygous recessive disease.** Star marked Individuals were not sequenced. Individuals in grey in the pedigrees had unknown phenotype. Individuals with no color are unaffected. Individuals in black color are affected with hypoplasia of cerebellum in **A**, and with abnormal pain sensation in **B**.

1. 108 unrelated individuals from Qatar
  Genotypes from HumanOmni2.5-8 array and Whole Genome Sequencing (WGS) data analysis from CASAVA and GATK pipeline are compared for these 108 unrelated individuals from Qatar. Whole Genome Sequencing was done by Illumina Hiseq 2500 sequencer with the average coverage of 37.99 (see Additional file 1). Phenotypes of these individuals are either diabetic or normal individuals. Illumina CASAVA pipeline called variants using a single sample (genome) at a time while we used GATK multi-sample calling.
2. 19 trios (Father, Mother, Offspring)
  These 19 trios are selected from another cohort of 64 individuals with 11 different families. Whole Genome Sequencing was done by Illumina Hiseq 2500 sequencer with the average coverage of 39.50 (Additional file 1). Variants from the GATK pipeline were called for all the 64 individuals together by multi-sample calling using the UnfiedGenoytper and variant sets for trios were filtered using SelectVariants walker. The pedigree structures for families from which trios are selected are shown in Figure 1.
3. Two clinical case studies of monogenic disorders
  In the first family (Figure 2A), both affected children are present with hypoplasia of cerebellum as a disease phenotype. The other three members (Father, mother and aunt) are unaffected. The second family (Figure 2B) comprises two affected children with abnormal pain sensation and two unaffected children. Whole Genome Sequencing was done by Illumina Hiseq 2500 sequencer with the average coverage of 43.20 for first family and 42.95 for second family (Additional file 1). In the pipeline comparison result for these two families, GATK calls were made by multi-sample calling of all members of the family whereas CASAVA calls were from single sample calling.

For all three evaluation data sets, although Illumina supplied annotated VCF files, we annotated both Illumina and GATK VCF files using SnpEff [21] and AnnTools [22] to provide a uniform annotation for comparison between pipelines.

## Results

The summarized comparison results between the CASAVA and GATK pipeline are presented in Table 1. Both CASAVA and GATK have very high similarity to OmniArray genotypes. However, while comparing all variants from NGS, GATK identifies a higher number of variants than CASAVA. The robustness of these additional variants are analyzed and discussed below in the results presented for comparison between the pipelines for various data sets.

**Table 1:** **Summary of GATK and CASAVA comparison**

Data sets			CASAVA		GATK		Common	
			Variant count	TsTv ratio	Variant count	TsTv ratio	Variant count	TsTv ratio
**Omni Array Genotyping Data**								
108 unrelated (per sample)	All	SNP by pipelines	708,089 ± 4,516	3.56 ± 0.003	715,033 ± 4,551	3.53 ± 0.003	706,378 ± 4502	3.57 ± 0.003
		GT Matched SNP	705,749 ± 4468	3.58 ± 0.003	703,608 ± 4479	3.59 ± 0.003	698,910 ± 4437	3.60 ± 0.003
		Ref or missing by pipeline	1661347 ± 4538		1654613 ± 4573		1649566 ± 4567	
		False positive	988 ± 20	1.46 ± 0.020	6320 ± 112	1.61 ± 0.039	489 ± 13	1.92 ± 0.031
		False negative	23283 ± 90		22076 ± 58		20550 ± 59	
	PASS	SNP by pipelines	707,128 ± 4506	3.57 ± 0.003	696,960 ± 4480	3.60 ± 0.003	695,589 ± 4456	3.60 ± 0.003
		GT Matched SNP	705,106 ± 4459	3.58 ± 0.003	693,135 ± 4434	3.63 ± 0.003	689,330 ± 4394	3.63 ± 0.003
		Ref or missing by pipeline	1662325 ± 4528		1672894 ± 4501		1657868 ± 4533	
		False positive	810 ± 17	1.33 ± 0.019	277 ± 20	2.23 ± 0.054	229 ± 10	2.12 ± 0.054
		False negative	24067 ± 134		33985 ± 96		22735 ± 65	
**NGS Data Set**								
108 unrelated (per sample)	All		4,025,625 ± 44,102	2.02 ± 0.001	4,331,336 ± 45,896	1.86 ± 0.002	3,792,293 ± 43,122	2.07 ± 0.000
	PASS		3,894,810 ± 43,388	2.04 ± 0.001	3,438,203 ± 41205	2.13 ± 0.001	3,401,091 ± 40317	2.15 ± 0.001
19 trios ( per trio)	ALL		5,235,184 ± 47,790	2.01 ± 0.001	7,003,439 ± 56,488	1.88 ± 0.003	4,945,042 ± 46,650	2.06 ± 0.002
	PASS		4,786,871 ± 47,564	2.07 ± 0.002	5,125,002 ± 48,717	2.13 ± 0.001	4,320,414 ± 43,062	2.14 ± 0.001
Family 1	ALL		6,082,624	2.00	6,337,108	1.88	5,635,183	2.04
	PASS		5,438,393	2.07	5,004,048	2.12	4,898,126	2.13
Family 2	ALL		5,192,891	1.99	5,459,725	1.84	4,752,193	2.03
	PASS		4,526,291	2.07	4,205,995	2.12	4,104,343	2.13

Comparison between the pipelines have been done for unfiltered sets (CASAVA ALL, GATK ALL), and for quality filtered sets (CASAVA PASS, GATK PASS).

### Comparison of NGS pipelines with genotyping array

The Illumina Omni 2.5 platform can detect genotypes at 2.37 million SNP loci in the human genome. In every single individual about 30% of these 2.37 million SNPs were present either in a heterozygous or a homozygous for the non-reference variant state. Illumina only reports genotypes for such variants in the VCF files. Reference allele homozygous calls are not differentiated from non-call. We therefore compare the pipeline only on SNPs that are reported in the VCF files. Both pipelines have very high concordance (~99%) with genotyping array data (Table 1). GATK pipeline has a higher number of non-reference SNPs compared to CASAVA, but CASAVA has slightly higher genotyping matches (99.67%) compared to GATK (98.33%). For quality passed variants (CASAVA PASS, GATK PASS) both pipelines have approximately the same concordance with Illumina Genotyping OmniArray data (Table 1 and Additional file 2). False positives and false negatives in Table 1 are calculated assuming Illumina OMNI 2.5 genotype data to be correct. GATK has lot more false positive compared to CASAVA before PASS filter and the opposite after PASS filter. To our surprise, TsTv ratios of these false positives are not very far from ideal TsTv ratio of 2.0-2.1. Furthermore, TsTv ratio of false positive by GATK is better, closer to 2, than the TsTv ratio of false positive by CASAVA in both before and PASS filter. Moreover, the TsTv ratio of common false positive is near to 2 suggesting these small numbers of common false positive by both pipelines could be false negative in OMNI 2.5 genotype array data.

### Pipeline comparison in unrelated individuals

**Figure 3:**
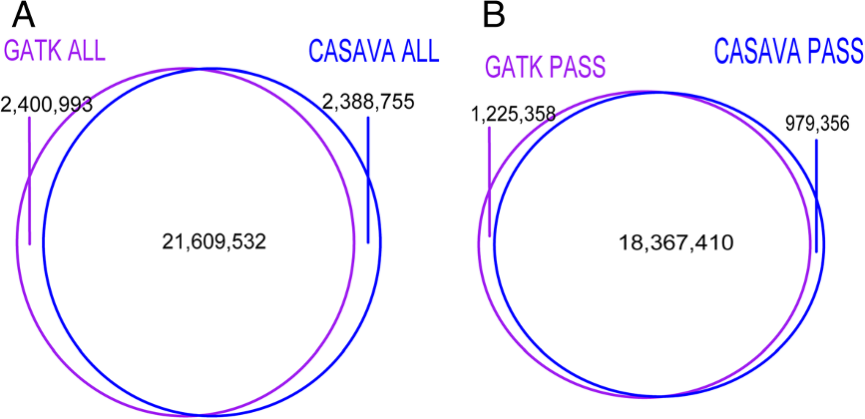
**Venn diagrams comparing combined variants of All108 Qatari genomes by GATK and CASAVA pipelines. A)** Between GATK ALL and CASAVA ALL variants sets showing intersection and unique variants sets. **B)** Between GATK PASS and CASAVA PASS variants sets showing intersection and unique variants sets.

**Figure 4:**
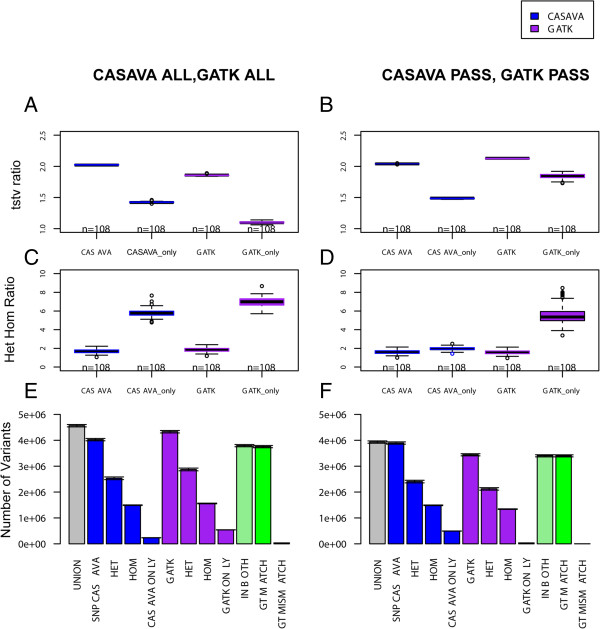
**Individual genome comparison between GATK and CASAVA pipeline in 108 unrelated Qatari individuals. A**, **C,** and **E** show comparison for unfiltered sets (CASAVA ALL, GATK ALL). **B**, **D**, and **F** show comparison for quality filtered sets (CASAVA PASS, GATK PASS). **A** and **B** show boxplots of 108 transition-transversion (TsTv) ratios for pipeline’s variants sets (CASAVA, GATK) along with pipeline specific (CASAVA ONLY, GATK ONLY). C and D show boxplots of 108 het-hom ratios for pipeline’s variants sets (CASAVA, GATK) along with pipeline specific (CASAVA ONLY, GATK ONLY). E and F show barplot of average variant counts for 108 individuals with error at the top of each bar.

**Figure 5:**
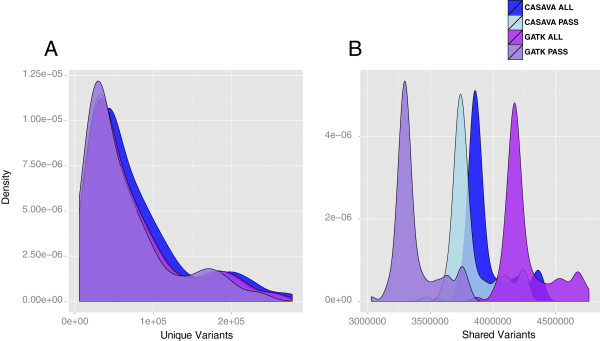
**Shared and unique variants by GATK and CASAVA in 108 unrelated Qatari individuals. A** shows density plot of unique variants of 108 unrelated Qatari individuals in GATK ALL, GATK PASS, CASAVA ALL, and CASAVA PASS variant sets. **B** shows density plot of shared variants of 108 unrelated Qatari individuals in GATK ALL, GATK PASS, CASAVA ALL, and CASAVA PASS variant sets.

Venn diagram in Figure 3 shows the comparison between CASAVA and GATK pipeline for the combined variants of all 108 unrelated individuals. For the unfiltered variants set in Figure 3A, GATK ALL and CASAVA ALL have an approximately equal number of SNPs (24.01 million for GATK and 23.99 million for CASAVA) and an equal number of unique SNPs (2.4 million for GATK and 2.39 million in CASAVA). However, if we look at the individual sample from GATK and CASAVA in Figure 4E, we find GATK has many more SNP calls than CASAVA (4.33 million by GATK and 4.02 million by CASAVA). This discrepancy, similar number of variants by pipelines at population level but different at sample level, can be explained by exploring shared and unique variants across the samples (Figure 5). The number of shared variants among 108 individuals identified by GATK is lot more than in CASAVA for both with and without PASS filter (Figure 5a, Figure 5b). The distribution of number of unique variants among 108 individuals identified by GATK and CASAVA are overlapping in great extant and thus are very similar (Figure 5c and Figure 5d). Also, we can explain the discrepancy by pipelines at population and sample level by looking at the pipeline specific calls (GATK ONLY and CASAVA ONLY calls). Theoretically, CASAVA ONLY calls should be very different across the 108 samples and GATK only calls should be similar across 108 samples to justify the observed discrepancy. When we checked the GATK ONLY 2.4 million SNPs of combined variants set (Figure 3A), we found that around 56.6% (1.29 million) were present in more than 5 out of 108 samples. In contrast, in CASAVA ONLY 2.39 million combined variants (Figure 3A), only 18.8% (0.45 million) were present in more than 5 out of 108 samples. The higher percentage of consensus call across the sample in GATK ONLY SNPs compared to CASAVA ONLY SNPs indicates the effects of multi-sampling calling using the GATK pipeline. We hypothesize that this effect is desired since the samples are from the same population. In other words, in order to have confidence in the SNPs that are non-agreeing across the pipelines, the variant calls should have agreement across the samples, provided that the samples originate from same population. However, the variants identified by only one pipeline (GATK ONLY SNPs or CASAVA ONLY SNPs) have lower TsTv ratio compared to variants that are common to both pipeline (Figure 4A and Figure 4B). TsTv ratio for GATK ONLY SNPs before pass filter in Figure 4A is very low (1.096 ± 0.003). Similarly, TsTv ratio of CASVA ONL SNPs in Figure 4B is low (1.485 ± 0.001). The lower TsTv ratio of pipeline specific variants indicates the presence of false positives. Furthermore, Het/Hom ratio of GATK ONLY subset after GATK PASS filter is very high, as shown in Figure 4D, indicates that GATK calls more false positive heterozygous calls than homozygous false positive calls. In general, the explanation of lower TsTv for both before and after PASS filter should be similar. The more number of pipeline specific variants has more false positives. In addition to the pipeline specific variant count, the lower quality variants could be reason of of very low Tstv ratio for GATK ONLY in Figure 4A compared to TsTv ratio of GATK ONLY subset in Figure 4B. However, before pass filter the number of combined set of variants for GATK ONLY (2.4 million) is similar to CASAVA ONLY (2.39 million) and, therefore, should not have drastically different TsTv in data set. Moreover, GATK ONLY subset has more number of shared variants among 108 samples compared to CASAVA ONLY and intuitively we would be expecting better TsTv for GATK ONLY compared to CASAVA ONLY. The opposite behavior of TsTv can thus be attributed to GATK multi-sample calling which might be placing doubtful SNP in samples at particular locus if it one or more samples have confirmed SNP at that locus. This suggests that multi-sample calling has the advantage of calling more variants but at the cost of more false positives. The other possible explanation of lower TsTv ratio of pipeline specific variants could be non-universal nature of TsTv ratio [23]. However, we tested this by random sampling the 2.4 million variants 10 times and computed TsTv ratio. We found TsTv ratio of these randomly sampled variant to be 2.051 ± 0.001. This excludes non-universal nature of TsTv ratio as possible explanation. Thus, lower TsTv for pipeline specific (GATK ONLY and CASAVA ONLY) subset is indication of false positives. The non-agreeing SNPs between the pipelines can also be analyzed in a family structure to see the Mendelian violation, which we did by looking at 19 trios (Father, Mother, and Offspring) and 2 families having homozygous recessive diseases.

Pipeline differences after PASS filter at per sample level (Figure 4F) is apposite to before PASS filter (Figure 4E) i.e. the number of SNPs per sample in GATK call set is lower than in CASAVA. However, at population level GATK called more SNPs in both before and after PASS filter (Figure 3A and Figure 3B). It is important to see how PASS filter changed the allele frequency distribution in GATK and CASAVA. Minor Allele Frequency (MAF) distribution plot shown in Additional file 3 and variants frequency distribution shown in Additional file 4 to see the effect of PASS filter for both GATK and CASAVA. In Additional file 3, we can see that PASS filter removes low frequency with high MAF and, therefore, we see higher frequency for low MAF. In Additional file 4, we can see the distributions of GATK before and PASS filtering is far apart while the distribution of CASAVA before and PASS filtering has some overlap. This shows that there are many low quality variants from each of the 108 unrelated samples identified by GATK. This also explains the reason of higher false positives and lower TsTv ratio for of GATK compared to CASAVA before PASS filter.

### Pipelines comparison in trios

**Figure 6:**
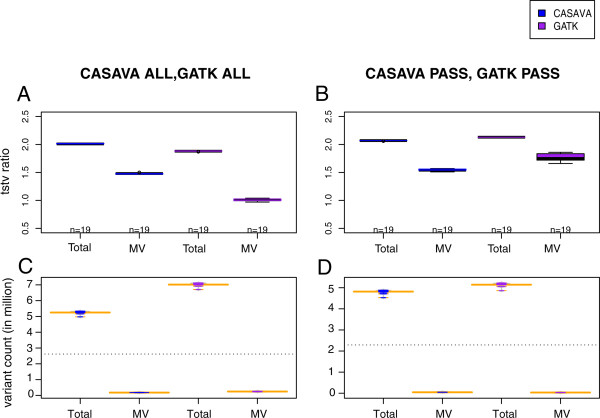
**GATK and CASAVA comparison in 19 trios. A** and **C** show comparison for unfiltered sets (CASAVA ALL, GATK ALL). **B** and **D** show comparison for quality filtered sets (CASAVA PASS, GATK PASS). A and B shows boxplots of 19 transition-transversion ratios for pipeline’s variants sets (Total_TsTv) along with TsTv of varaints with Mendelian violations. C and D shows beanplots of 19 trios total and Mendelian violated variant sets.

The CASAVA and GATK pipelines were compared for 19 trios from the Qatari population by taking combined variants sets of each trio separately (Figure 6 and Additional file 5). On average GATK ALL have 7 million variants in any trio compared to 5.25 million variants in CASAVA ALL (Figure 6). The large difference between the GATK ALL and CASAVA ALL variant sets in any trio can be attributed to GATK multi-sample calling, but this gives rise to the question about the qualities of these extra variants. Both pipelines have approximately equal percentage of variants having Mendelian violation (3.40% for CASAVA ALL and 3.47% for GATK ALL (Figure 6C). Assuming Mendelian violation as a criterion to judge confidence in variants, CASAVA pipeline missed those extra 1.75 million variants present in GATK ALL, which were comparable in quality. However, the lower TsTv ratio of 1.01 for Mendelian violated GATK ALL variants compared to TsTv ratio of 1.47 for Mendelian violated CASAVA ALL variants (Figure 6A) creates doubt about these extra 1.75 million variants of GATK ALL. The difference between number of variants, Mendelian violation, and TsTv ratio in the GATK and CASAVA pipelines is diminished for quality-filtered sets (CASAVA PASS and GATK PASS). It’s important to estimate false positive rate for the decision of applying the PASS filter or not. Since we do not have genotyping array data for trios, we are confined to assess the pipelines performance based on Mendelian violation and TsTv ratio. We can assume the variant set confirmed by both pipelines to be robust to provide us the rough estimate of the Fraction of Mendelian violation (F_MV_) in each trio by the pipeline. Using this fraction we thus computed the Expected number of Mendelian violation in variants subset (E_MV_) which didn’t pass the PASS filter (NOT PASS) in the pipelines. We then found the actual number of variants with Mendelian violation (O_MV_) in all the variants in NOT PASS subset (T_NP_). We then calculated the false positive fraction in the NOT PASS subset by (O_MV_ – E_MV_)/T_NP_. This way we found mean false positive percent of 11.15% for GATK NOT PASS subset and 22.90% for CASAVA NOT PASS subset in 19 trios. The detailed numbers are shown in Additional file 6. The false positive percent in CASAVA NOT PASS is higher than GATK NOT PASS in trios that is in contrast to false positive percent by pipelines in 108 unrelated individuals when computed using Genotyping OMNI array data. It suggests that GATK multi-sample calling algorithm works better in related individuals compared to unrelated individuals. In order to check the real difference at a functional level, we evaluated the pipeline performance in real case of finding the causative homozygous recessive variant in two diseased families.

### Pipelines comparison for calling variants in monogenic homozygous recessive diseased families

We analyzed two different families with affected children. In Family 1, affected children were diagnosed with the phenotype of hypoplasia of cerebellum which is monogenic homozygous recessive disease [24–28]. In Family 2, affected children were diagnosed with abnormal pain sensation, which is also monogenic homozygous recessive disease [29–33]. The number of variants between pipelines and between quality filter sets follows the similar pattern of what we saw above in the comparison between the pipelines in trios. However, in these cases, the difference in Mendelian violation between the pipelines is strongly pronounced. The difference is more between CASAVA ALL and GATK ALL variants set and the detail of this shown in Table 2 and Table 3 for Diseased Family 1 and Diseased Family 2 correspondingly. Since, CASAVA PASS and GATK PASS variants sets have lot of similarity; the details of number of variants for various categories are shown in Additional file 7 and Additional file 8 for Diseased Family 1 and Disease Family 2 correspondingly.

**Table 2:** **GATK and CASAVA comparison in diseased Family 1**

Pipelines	CASAVA all			GATK all		
		HRC			HRC	
	Total	Total	HRC by CASAVA but not by GATK	Total	Total	HRC by GATK but not by CASAVA
Total number of variant	6082624	61497	409	6337108	63774	1349
TsTv Ratio	2.00	2.09	1.88	1.88	2.03	1.07
Mendelian Violation	290985	0	0	187920	0	0
In dbSNP	5562308	60769	268	5609116	62277	643
In interGenic	3682138	35416	257	3790849	36884	846
In CDS	65785	650	2	64452	638	2
In ‘3 UTR	77701	849	16	82211	853	11
In ‘5 UTR	18552	240	0	20093	246	4
In Intronic	4113210	47220	218	4412928	48805	818
In Non_coding_intronic	214239	1826	26	217104	1839	40
In Exonic	57842	580	1	56762	579	3
In Non_coding_exonic	16356	110	0	15196	106	0
In Putative Promoter Region	12780	135	0	15517	141	1
Non-Synonymous Coding	19080	159	1	18299	161	2
Common Variant	5395888	60389	205	5905677	62830	1335
Common Homozygougs Minor Variant in 1000genome	4777175	56874	104	4792920	57620	194
Common Homozygougs Minor Variant in Q108	4727231	57643	142	4915274	60169	1293
Common Het (>5%) Variant in 1000genome	4479609	56407	99	4494483	57151	189
Common Het(>5) Variant in Q108	4895168	59296	63	5422841	62027	1332

Comparison between the pipelines have been done for unfiltered sets (CASAVA ALL, GATK ALL), and subset of variants fulfilling the criteria of Homozygous Recessive Conditions (HRC).

**Table 3:** **GATK and CASAVA comparison in diseased Family 2**

Pipelines	CASAVA all						GATK all					
		HRC						HRC				
			HRC by CASAVA but not by GATK						HRC by GATK but not by CASAVA			
	Total	Total	Total	GT mismatch by GATK		Absent in GATK	Total	Total	Total	GT mismatch by CASAVA		Absent in CASAVA
Total number of variant	5192891	29522	912	781		131	5459725	30653	2043	1499		544
TsTv Ratio	1.99	2.04	1.30	By CASAVA	1.21	2.05	1.84	2.00	1.29	By CASAVA	1.37	1.09
				By GATK	1.23					By GATK	1.37	
Mendelian Violation	416915	3	1	By CASAVA	0	1	265402	8	8	By CASAVA	1010	5
				By GATK	244					By GATK	3	
Mendelian Violation in affected	279336	0	0	By CASAVA	0	0	189043	0	0	By CASAVA	929	0
				By GATK	211					By GATK	0	
Mendelian Violation in unaffected	267367	3	1	By CASAVA	0	1	182659	8	8	By CASAVA	257	5
				By GATK	82					By GATK	3	
In dbSNP	4717672	28409	738		656	82	4754687	29129	1458		1243	215
In interGenic	3152905	18780	602		516	86	3266794	19527	1349		999	350
In CDS	54766	339	6		3	3	53607	359	26		11	15
In ‘3 UTR	64961	476	4		4	0	69395	483	11		8	3
In ‘5 UTR	15087	81	0		0	0	16635	92	11		10	1
In Intronic	3484737	19668	487		427	60	3797577	20562	1381		976	405
In Non_coding_intronic	180421	931	38		29	9	182875	972	79		57	22
In Exonic	48608	284	6		3	3	47577	292	14		7	7
In Non_coding_exonic	13960	82	8		8	0	12525	82	8		7	1
In Putative Promoter Region	10932	56	0		0	0	13801	66	10		10	0
Non-Synonymous Coding	16299	97	4		3	1	15429	100	7		3	4
Common Variant (1000genome + Q108)	4693812	28034	784		728	56	5224846	29186	1936		1418	518
Homozygougs Minor Variant in 1000genome	4089468	26162	396		364	32	4098301	26562	796		726	70
Homozygougs Minor Variant in Q108	4283672	26479	663		628	35	4469485	27677	1861		1357	504
Het (>5%) Variant in 1000genome	3888895	25824	386		354	32	3897416	26225	787		717	70
Het(>5) Variant in Q108	4355312	27616	686		674	12	4900523	28852	1922		1408	514
Non-Synonymous rare Pain related	5	1	0		0	0	5	1	0		0	0
Pain genes mapped to Non-Synonymous rare variants	4	1	0		0	0	4	1	0		0	0

Comparison between the pipelines have been done for unfiltered sets (CASAVA ALL, GATK ALL), and subset of variants fulfilling the criteria of Homozygous Recessive Conditions (HRC).

#### Diseased family 1

The CASAVA pipeline has 4.78% (290985 out of 6082624) of variants with Mendelian violation in the CASAVA ALL set and 1.23% (66903 out of 5438393) of variants with Mendelian violation in the CASAVA PASS set. In contrast, GATK has only 2.96% (187920 out of 6337108) of variants with Mendelian violation in the GATK ALL set and 0.14% (7122 out of 5004048) of variants with Mendelian violation in the GATK PASS set (Table 2 and Additional file 7). Because both children are affected by the hypoplasia of cerebellum, and the parents and aunt are unaffected, the causative variant should be a homozygous variant [34]. We further investigated the pipeline performance to find the homozygous recessive variants. In this paper, we use the term Homozygous Recessive Condition (HRC) for any particular variant position in a family when all three of the following conditions are met: 1) all affected off-springs are homozygous, 2) all affected off-springs have the same genotype and their genotype is different than normal individuals in the family, and 3) all affected off-springs follow Mendelian inheritance (e.g. Father GT = A/C, Mother GT = A/C, Affected Child 1 GT = C/C, Affected Child 2 GT = C/C). Both CASAVA and GATK pipelines have approximately a similar number of HRC variants (Table 2). They also have a similar number of region specific or known variants like exonic, CDS, 3’UTR, 5’UTR, intronic, non-synonymous coding, 1000genome and so on. Furthermore, the pipelines have a similar number of commonly known variants such as those in 1000 genomes, and Q108 (108 unrelated individuals from Qatar). After filtering the known variants, we tried to map these variants to known genes for the phenotype in the literature. We could not map the set of possible causative variants to known genes in this case. Therefore, we tried another real case of homozygous recessive disease with a pair of normal and affected siblings.

#### Diseased family 2

This family is different in structure because of the presence of unaffected siblings (Figure 2B), which gives extra power to evaluate the pipeline because of the inherent validation about the variants, e.g. evaluating homozygous recessive variants identified by both the pipelines but mismatched in genotypes according to Mendelian inheritance in affected and unaffected separately. We have presented a detailed comparison of the pipeline performances for this family in Table 3. The additional parameters to judge the pipelines in Table 3, as compared to the previous case in Table 2, are due to the additional two normal siblings in this case. Exclusively determined HRC variants are divided into two sets of variants for analyzing pipeline performance: 1) HRC variant by Pipeline1 and not by Pipeline2 and having mismatch in genotype calls between the pipelines, and 2) HRC variant by Pipeline1 and none from Pipeline2 for all five individuals.

In the first set of variants (Table 3, Column "GT mismatch by GATK" and "GT mismatch by CASAVA"), in which only one pipeline meets HRC and the pipelines have mismatch in genotype calls, the pipeline not meeting HRC can either have Mendelian inheritance or Mendelian violation. The cases, where both the pipelines have Mendelian inheritance and only one pipeline meets HRC, are difficult to evaluate in terms of pipeline performance. Example variant position genotypes in this family are as follows:

*Pipeline 1 Genotypes (Mendelian Inheritance and HRC):* Father GT = A/C, Mother GT = A/C, Unaffected Child1 GT = A/C, Unaffected Child2 GT = A/C, Affected Child1 GT = C/C, Affected Child1 GT = C/C;

*Pipeline2 Genotypes (Mendelian Inheritance but no HRC):* Father GT = A/A, Mother GT = A/C, Unaffected Child1 GT = A/A, Unaffected Child2 GT = A/A, Affected Child1 GT = A/C, Affected Child2 GT = A/C.

The cases where one pipeline has both HRC and Mendelian inheritance and other pipeline has neither could be a strong indication that the second pipeline calls are wrong in these variants. Example variant position genotypes in this family are as follows:

*Pipeline 1 Genotypes (Mendelian Inheritance and HRC):* Father GT = A/C, Mother GT = A/C, Unaffected Child1 GT = A/C, Unaffected Child2 GT = A/C, Affected Child1 GT = C/C, Affected Child1 GT = C/C;

*Pipeline2 Genotypes (No HRC due to Mendelian violation in affected off-springs):* Father GT = A/A, Mother GT = A/A, Unaffected Child1 GT = A/A, Unaffected Child2 GT = A/A, Affected Child1 GT = C/C, Affected Child1 GT = C/C.

In Table 2, we can see in the CASAVA ALL and GATK ALL sets that out of 1499 exclusively determined HRC variants by GATK, 929(62%) had both Mendelian violations and different genotypes by the CASAVA pipeline. In contrast, out of 781 exclusively determined HRC variants by CASAVA, only 244 (31%) have both Mendelian violations and different genotypes. Therefore, we can say that for exclusively determined HRC where there is mismatch between the genotype calls between the pipelines, the GATK pipeline is more robust than the CASAVA pipeline, if we compare all the variants without any quality filter.

We also examined Mendelian violation in another set of exclusively determined HRC variants by one pipeline where there were no variants in any member of the family by the second pipeline (Table 3, Column "Absent in GATK" and "Absent in CASAVA"). Both CASAVA and GATK have almost no Mendelian violation in these cases.

Table 3 also shows many categories to compare CASAVA and GATK. CASAVA identifies slightly more number of Non-synonymous variants compared to GATK. However, GATK has higher percentage of Non-synonymous variants as HRC variants compared to CASAVA. About one hundred of these Non-Synonymous variants of both the pipelines are linked to 60 pain related genes by literature identified using SnpEff [21] and AnnTools [22]. After excluding the common variants (variants present in homozygous state in either 1000 genomes or 108 unrelated Qatari individuals, and variant present in heterozygous state with MAF >5%) from these non-synonymous variants, there were 5 variants left by both the pipelines (Non-synonymous pain related rare variant in Table 3). From both pipelines, out of these 5 variants only one was HRC variant and most probably the causative variant.

## Discussion

We found excellent performances of both GATK and CASAVA pipelines in matching the genotype calls when matching with Illumina OmniArray genotype calls. However, we saw differences in the number of variants called by each pipeline in unfiltered variant sets (CASAVA ALL, GATK ALL) and generally GATK identifies more variants because of its multi-sample calling algorithm. Most of these additional variants are of low quality but not bad in terms of Mendelian inheritance. CASAVA pipeline, in most of the cases, have TsTv ratio closer to 2 compared to GATK. Since both CASAVA and GATK pipeline were unaware of the pedigree structure while calling the genotypes, in conflicting or discordant genotypes by the pipelines, Mendelian inheritance is a good criterion to judge the confidence of variants for familial samples. In general, GATK pipeline called less Mendelian violation for all different sets. Notably, PASS filter in GATK pipeline drastically minimizes Mendelian Violation, from 2.4% in GATK ALL to 0.14% in GATK PASS in disease family 1 and from 4.86% in GATK ALL to 0.19% in GATK PASS in disease family 2. However, in CASAVA pipeline PASS filter does not reduce Mendelian violation significantly, from 4.78% in CASAVA ALL to 1.23% in CASAVA PASS in disease family 1 and from 8.03% in CASAVA ALL to 1.87% in CASAVA PASS in disease family 2. By assuming Mendelian violation to be inversely correlated to pipeline performance in cases of genotype mismatch and where the other pipeline satisfies HRC, GATK multi-sample calling performs better than CASAVA single sample calling for these cases. However, we didn’t find any significant difference in the ability of these pipelines to identify causative variants in this abnormal pain perception family. We also found extremely low Mendelian violation in exclusively determined homozygous recessive condition for which variants were not called in any family member by the other pipeline, which suggests robustness of both GATK and CASAVA pipelines in finding the functional variants. This broad level agreement between the pipelines suggests that normally we can avoid calling variants again using more sophisticated algorithm except for specific scientific goals. One of such specific scientific goals could be finding de novo mutation in samples where comprehensiveness of variants are desired and can be obtained by taking combining the variant sets from the pipelines with tolerated false positives. Also, if the cohort sample size is large and scientific goal is based on the phase SNPs, it is desirable to use more sophisticated SNP calling platform such as GATK multiple-sample calling.

On other note, the results presented here should hold for newer version of GATK as well. In furtherance, we did the sensitivity analysis (see Additional file 9) for 10 different versions of GATK released in last one and half year for our diseased family 2 data set. The relative standard deviation of variant counts of different versions of GATK for before and PASS filter sets are only 0.89% and 2.02% respectively while the difference between GATK and CASAVA presented in this paper using GATK v2.4 are around 4.9% and 7.6% before and PASS filter set respectively. Similarly, the relative standard deviation of TsTv of different versions of GATK for before and PASS filter sets are and only 0.58% and 0.59% respectively while the difference between GATK and CASAVA presented in this paper using GATK v2.4 are around 8.2% and 2.4% before and PASS filter set respectively. Thus, the different version of GATK have very little effect on the number of variants identified and thus doesn’t change the results and conclusion drawn in this paper using GATK v2.4.

We have used 3 different type of data set (108 unrelated, 19 trios, and 2 diseased families) to cover some of the various possible data sets. We have found difference in results for related and unrelated individuals. In general, the pipeline comparison results should hold for most of the possible data set with some limitations. We have only tested for sequences coming from Illumina platform that helps in fair comparison of the pipeline but the result might deviate for sequence reads from some other platform. Also, we have not tested for complex diseases like cancer where somatic mutation is frequent.

## Conclusion

High quality SNP calls delivered by commercial NGS sequencing projects in general show concordance with array genotypes and Mendelian inheritance. Application of more sophisticated SNP calling platforms, i.e. using GATK multiple-sample calling, may be helpful in validating and expanding the number of possible candidates, especially in related individuals, but may not provide additional candidates for monogenic disorders. In general, it is futile effort of calling variants again using open source alternative when commercial vendors had already supplied variants sets. However, in cases of related individuals where commercial providers lack the information of relatedness because of confidentially involved, one should try multiple-sample calling to expand variants conforming Mendelian inheritance.

## Electronic supplementary material

Additional file 1: **Coverage of samples.** (XLSX 34 KB)

Additional file 2: **Comparison of GATK and CASAVA pipeline with OmniArray.** (TIFF 657 KB)

Additional file 3: **Effect of PASS filter on Minor Allele Frequency distribution in 108 unrelated Qatari individuals.** (PDF 106 KB)

Additional file 4: **Effect of PASS filter on variant count distribution in 108 unrelated Qatari individuals.** (PDF 106 KB)

Additional file 5: **Het-hom of individual Father, Mother, and Off-spring in 19 trios with GATK and CASVA comparison.** (TIFF 685 KB)

Additional file 6: **Pipelines performances in 19 trios using Mendelian violation.** (XLSX 14 KB)

Additional file 7: **GATK and CASAVA comparison for quality filtered set in diseased Family 1.** (XLSX 12 KB)

Additional file 8: **GATK and CASAVA comparison for quality filtered set in diseased Family 2.** (XLSX 14 KB)

Additional file 9: **GATK version sensitivity analysis.** (XLSX 11 KB)

## Abbreviations

NGS: Next Generation Sequencing
WGS: Whole Genome Sequencing
SNP: Single Nucleotide Polymorphism
VCF: Variant Call Format
GT: Genotype
TsTv: Transition-transversion
CDS: Coding DNA Sequence
UTR: Un-translated Region
HRC: Homozygous Recessive Condition.
