# Supplementary figures and images for: Evaluation of SNP calling using single and multiple-sample calling algorithms by validation against array base genotyping and Mendelian inheritance
Source: BMC Res Notes. 2014 Oct 22;7:747. doi: 10.1186/1756-0500-7-747 (PMC4216909; doi:10.1186/1756-0500-7-747)

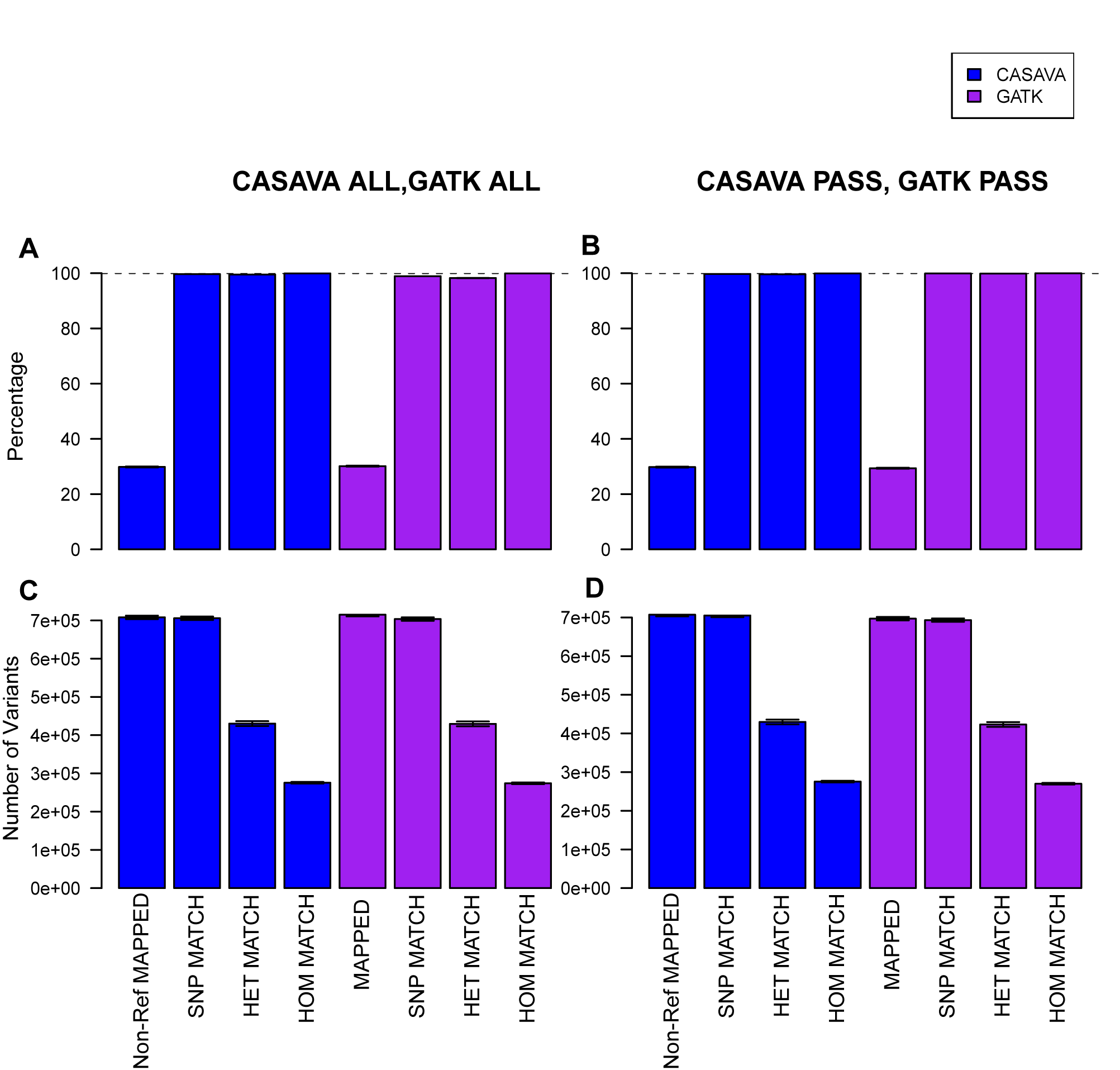

Supplement: Supplementary file 2 — Additional file 2: Comparison of GATK and CASAVA pipeline with OmniArray. (TIFF 657 KB) [file 13104_2014_3277_MOESM2_ESM.tiff]

**A**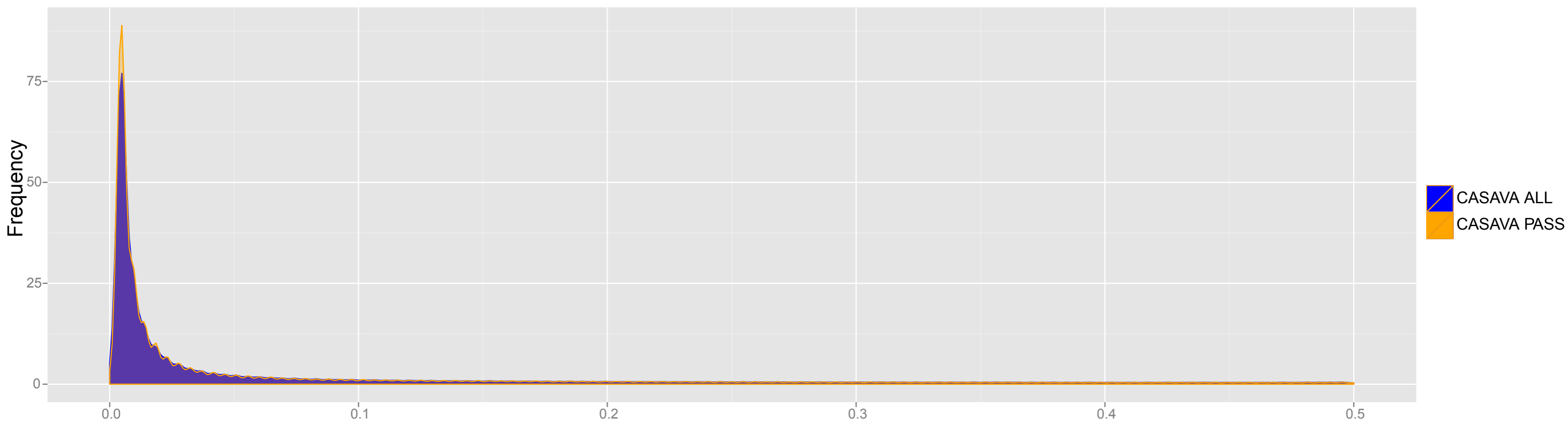**B**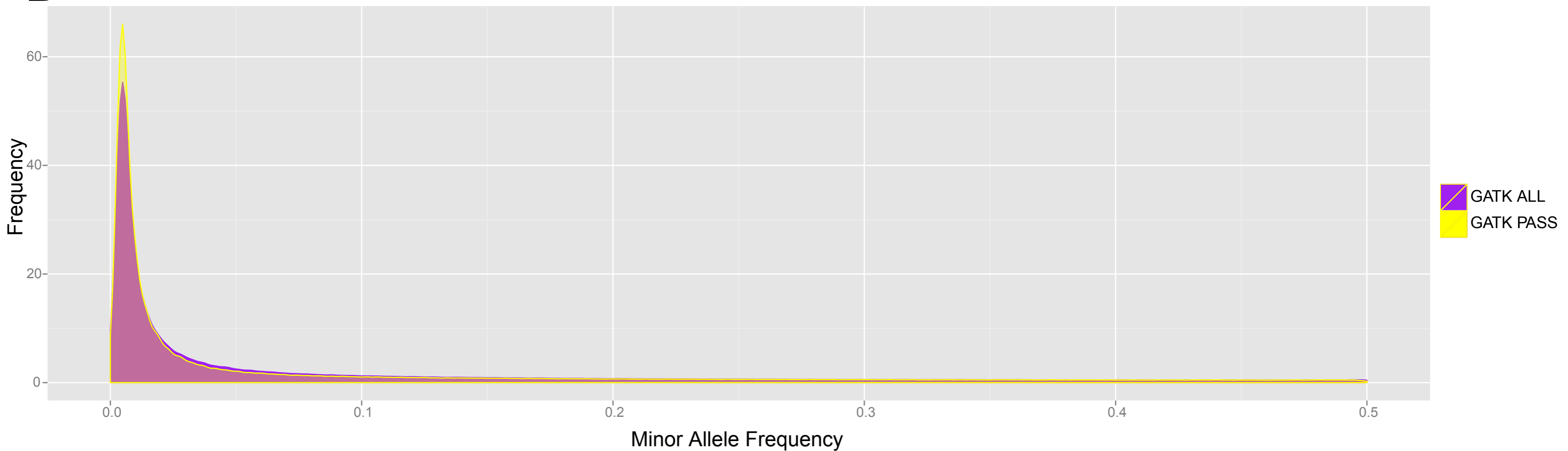

Supplement: Supplementary file 3 — Additional file 3: Effect of PASS filter on Minor Allele Frequency distribution in 108 unrelated Qatari individuals. (PDF 106 KB) [file 13104_2014_3277_MOESM3_ESM.pdf]

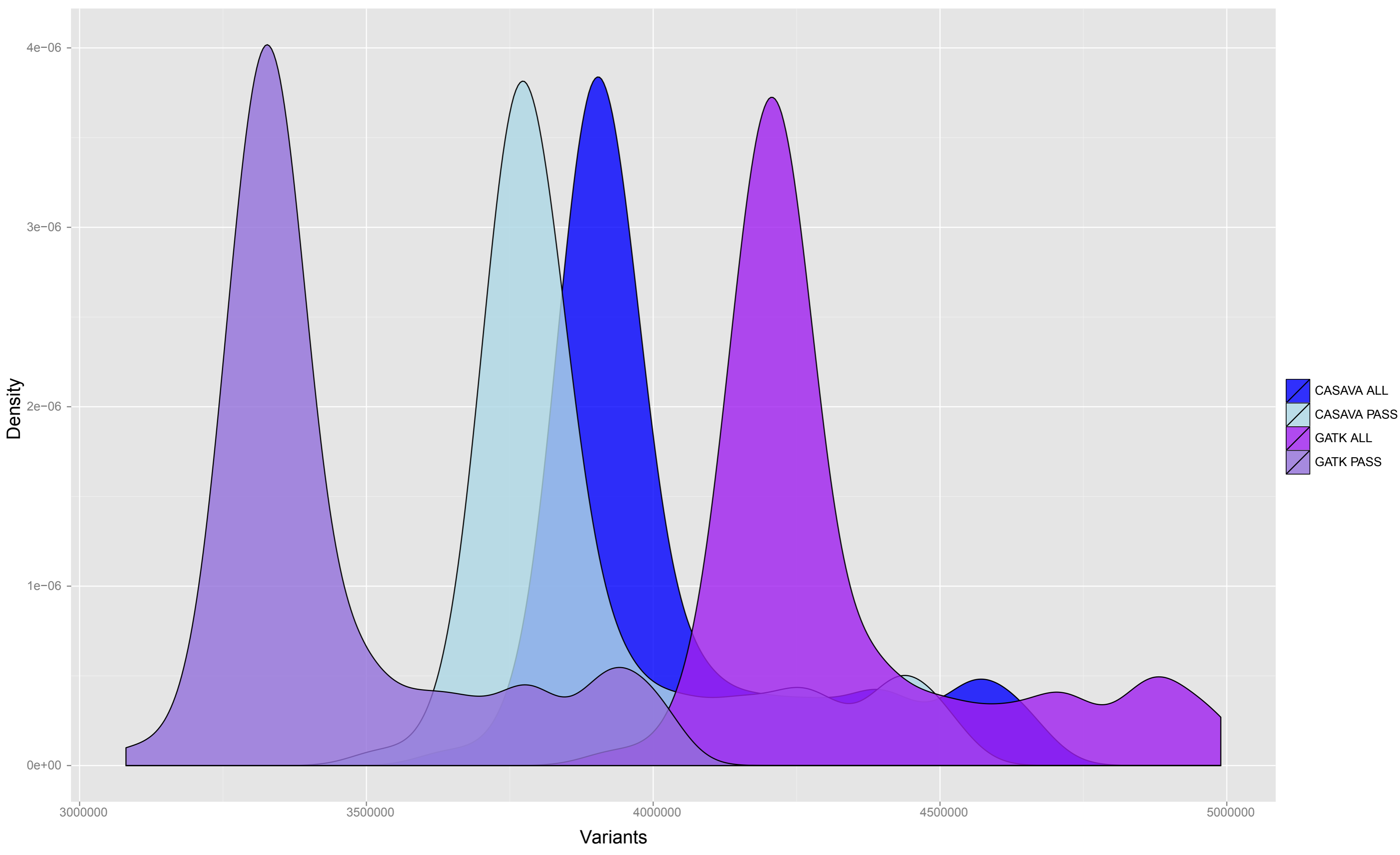

Supplement: Supplementary file 4 — Additional file 4: Effect of PASS filter on variant count distribution in 108 unrelated Qatari individuals. (PDF 106 KB) [file 13104_2014_3277_MOESM4_ESM.pdf]

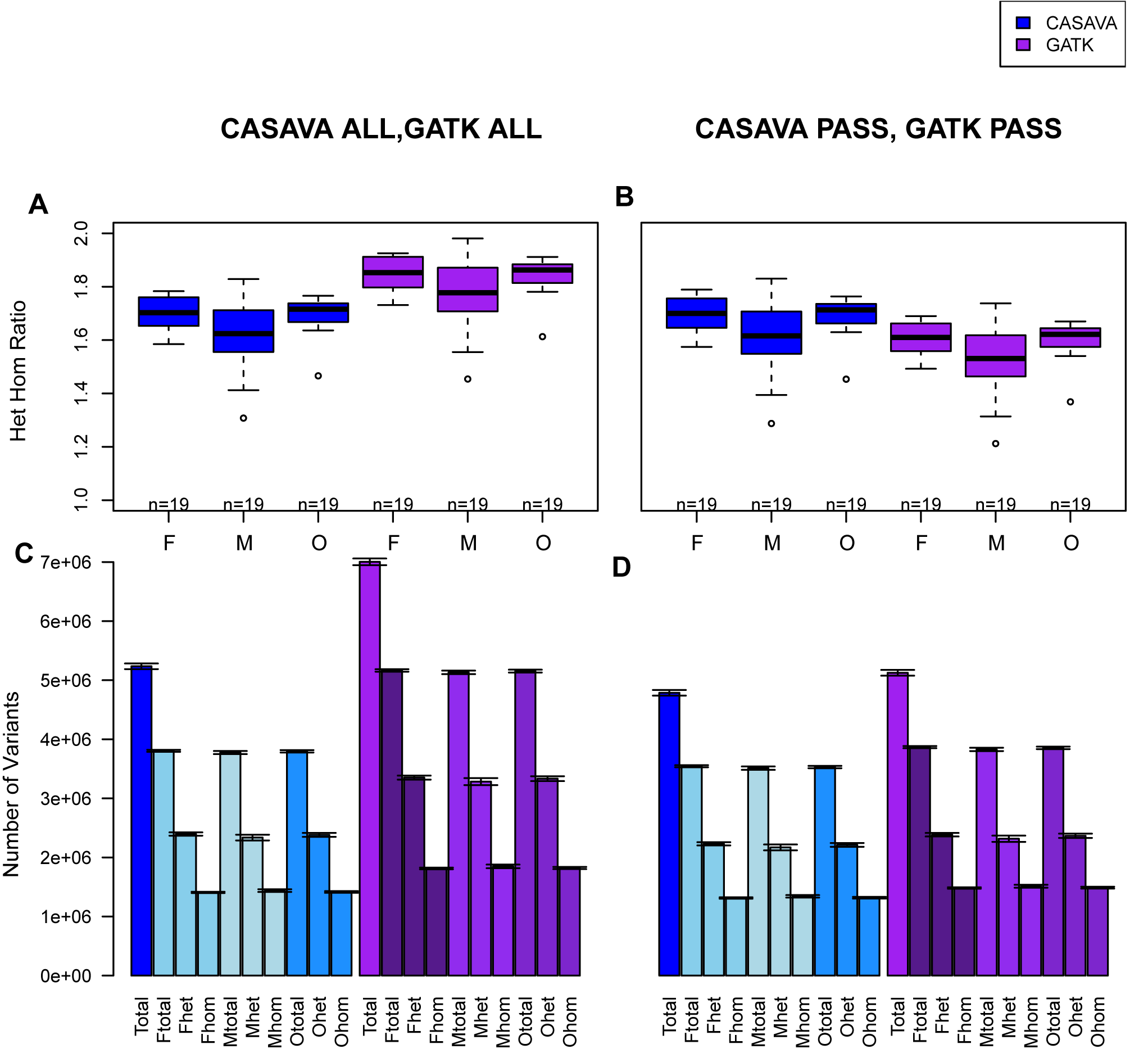

Supplement: Supplementary file 5 — Additional file 5: Het-hom of individual Father, Mother, and Off-spring in 19 trios with GATK and CASVA comparison. (TIFF 685 KB) [file 13104_2014_3277_MOESM5_ESM.tiff]
